# Physical exercise as a potential adjuvant therapy: effects on inflammation and nutrition in colorectal cancer patients—a systematic review and meta-analysis

**DOI:** 10.3389/fnut.2025.1612674

**Published:** 2025-06-26

**Authors:** Hui Zhang, Ying Zhang, Feifei Luo, Yixin Wen, Jiang Ni, Jing Wang

**Affiliations:** ^1^Department of General Surgery, The Fifth Hospital of Wuhan, Wuhan, China; ^2^Department of Nephrology, The Fifth Hospital of Wuhan, Wuhan, China; ^3^Department of Cardiac Function, Wuhan Fourth Hospital, Wuhan, China; ^4^Department of Orthopaedics, The Fifth Hospital of Wuhan, Wuhan, China; ^5^Department of Nutrition, The Fifth Hospital of Wuhan, Wuhan, China

**Keywords:** colorectal cancer, physical exercises, nutritional status, markers of inflammation, obesity

## Abstract

**Introduction:**

This meta-analysis aimed to reveal the effects of exercise training on markers of inflammation and indicators of nutrition in colorectal cancer patients.

**Methods:**

We systematically searched PubMed, Embase, Cochrane, and Web of Knowledge for randomized controlled trials (published between 1 January 1945 and 17 June 2024). Our main outcomes were nutritional status and markers of inflammation analyzed using a random-effects model.

**Results:**

Of the 3,081 identified studies, 15 were eligible and were included in our analysis (*N* = 996 participants). Compared with the usual care, physical exercise reduced C-reactive protein by a mean of −0.33 mg/dL (95% CI −0.62 to −0.04) in colorectal cancer patients. Similarly, body fat decreased by a mean of −1.36% (95% CI −2.52 to −0.99) after physical exercise. However, interleukin-6, tumor necrosis factor-alpha, and waist circumference were not different between patients who received physical exercise and those who received usual care. Subgroup analyses revealed that exercise duration ≤12 weeks was effective in decreasing body mass index of −0.59 kg/m^2^ (95% CI −1.15 to −0.02) and body weight of −3.12 kg (95% CI -4.66 to −1.58). In addition, body mass index (*p* = 0.005) and body weight (*p* = 0.03) were decreased in patients who combined aerobic exercise and resistance exercise.

**Conclusion:**

Overall, these findings suggest that physical exercise may improve inflammation status and enhance weight loss in CRC patients.

**Systematic review registration:**

Number CRD 42024536976.

## Introduction

Colorectal cancer (CRC) is the third most common cancer and the fourth leading cause of cancer-related death worldwide (1). China also faces a high CRC burden, accounting for approximately 38% of new global cases and 43% of global deaths (2). Beyond genetic factors, lifestyle and environmental risk factors significantly influence CRC development. Established risk factors include low-fiber/high-fat diets, sedentary behavior, obesity, smoking, alcohol consumption, and advanced age (3, 4). In recent years, rising CRC incidence has been linked to an aging population, dietary pattern changes, and increased prevalence of risk factors such as smoking, physical inactivity, and obesity (5). Chronic inflammation also plays a central role in carcinogenesis (6–9). For example, CRC incidence in inflammatory bowel disease patients is 50–60% higher than in the general population (10–12). Despite advancements in treatment, new therapies have had a limited impact on cure rates and survival. Consequently, there is a growing trend toward adjuvant strategies such as exercise (13).

Body mass index (BMI)-defined overweight (BMI ≥ 25–29.9 kg/m^2^) and obesity (BMI ≥ 30 kg/m^2^) is associated with multiple diseases. They represent the fifth leading risk factor for global mortality, causing at least 2.8 million adult deaths annually. Obesity, characterized by chronic low-grade inflammation, is a critical risk factor for colon cancer. Adipose tissue expansion correlates with elevated triglycerides, low-density lipoprotein (LDL) levels, and hyperinsulinemia—all presumed tumor-promoting mediators (14). Obesity also facilitates carcinogenesis via insulin/insulin-like growth factor 1 (IGF-1) pathway activation. Additionally, it increases proinflammatory cytokines (e.g., tumor necrosis factor-*α* [TNF-α], interleukin-1 [IL-1], and interleukin-6 [IL-6]) and alters adipokine profiles (15, 16). Meta-analyses show that colon cancer risk increases with higher BMI and waist circumference (17). CRC patients often exhibit poor physical function and exercise capacity, which elevate overall mortality and reduce quality of life, activities of daily living ability, and functional independence (4, 18, 19). Physical exercise is a core component of lifestyle modification for weight management and CRC progression control (20). Regular exercise reduces circulating IGF-1 levels, inhibits tumor cell proliferation, and induces autophagy or apoptosis (21). It suppresses abnormal angiogenesis by downregulating vascular endothelial growth factor-A (VEGF-A) in tumor tissues, thereby limiting the nutrient supply and metastatic pathways (22). Meanwhile, exercise promotes normal vascular remodeling, improves tumor tissue oxygenation, and enhances chemoradiotherapy sensitivity (23). Through multi-system, multi-target mechanisms, exercise remodels the tumor microenvironment—key effects include enhancing anti-tumor immunity, reversing metabolic abnormalities, inhibiting inflammation/angiogenesis, and regulating matrix mechanics (24). While the mechanisms remain incompletely understood, studies demonstrate that exercise effectively reduces inflammation and enhances immune function (4, 25). However, clinical practice has not clearly specified the specific details of exercise (such as type, duration, frequency, intensity, etc.), resulting in suboptimal effects of exercise interventions. Therefore, by analyzing how specific exercise characteristics impact the nutritional status, inflammatory markers, and quality-of-life indicators of CRC patients, this study aims to optimize current exercise-based adjuvant therapy methods.

Guided by this rationale, multiple clinical trials have evaluated routine care versus physical exercise in CRC patients. Recognizing that individual studies may lack sufficient power to inform practice, we aimed to objectively assess this intervention’s role in stages 1–4 CRC management. We therefore conducted a systematic review and meta-analysis of randomized controlled trials to quantify exercise effects on key outcomes: nutritional status and inflammatory biomarkers in CRC patients.

## Methods

### Search strategy and selection criteria

This systematic review and meta-analysis is reported by the Preferred Reporting Items for Systematic Reviews and Meta-Analyses (PRISMA) statement and was registered at the International Prospective Register of Systematic Reviews (number CRD 42024536976).

Two independent reviewers (HZ and FL) selected relevant studies published between January 1945 and June 2024 by searching PubMed Central, Embase, Cochrane, and Web of Knowledge on 17 June 2024, respectively. In addition, ongoing clinical trials about “physical exercise for CRC patients” were searched on the international clinical trial registry^1^ and conference abstracts as other sources (Supplementary Table S1). The correctness of the search string was then validated by JW. We applied no language restrictions. First, we searched PubMed for MeSH and entry terms on “colorectal cancer” and “physical exercise.” Then, concatenate their respective MeSH, entry terms, and abbreviations by the logical term “OR.” Finally, we connected the two searches using the logical term “AND,” which gave us the final search results. We used the following combined text and MeSH terms: “Colonic Neoplasms” and “Exercise.” The complete search used for PubMed was: (“Colonic Neoplasms” [MeSH Terms] OR Colonic Neoplasm [Text Word] OR Neoplasm, Colonic [Text Word] OR Neoplasms, Colonic [Text Word] OR Colon Neoplasms [Text Word] OR Colon Neoplasm [Text Word] OR Neoplasm, Colon [Text Word] OR Neoplasms, Colon [Text Word] OR Cancer of Colon [Text Word] OR Colon Cancers [Text Word] OR Colon Cancer [Text Word] OR Cancer, Colon [Text Word] OR Cancers, Colon [Text Word] OR Cancer of the Colon [Text Word] OR Colonic Cancer [Text Word] OR Cancer, Colonic [Text Word] OR Cancers, Colonic [Text Word] OR Colonic Cancers [Text Word] OR Colon Adenocarcinoma [Text Word] OR Adenocarcinoma, Colon [Text Word] OR Adenocarcinomas, Colon [Text Word] OR Colon Adenocarcinomas [Text Word] OR CRC [Text Word]) AND (“Exercise” [MeSH Terms] OR Exercises [Text Word] OR physical exercise [Text Word] OR Activities, Physical [Text Word] OR Activity, Physical [Text Word] OR Physical Activities [Text Word] OR Exercise, Physical [Text Word] OR Exercises, Physical [Text Word] OR Physical Exercise [Text Word] OR Physical Exercises [Text Word] OR Acute Exercise [Text Word] OR Acute Exercise [Text Word] OR Exercise, Acute [Text Word] OR Exercises, Acute [Text Word] OR Exercise, Isometric [Text Word] OR Exercises, Isometric [Text Word] OR Isometric Exercises [Text Word] OR Isometric Exercise [Text Word] OR Exercise, Aerobic [Text Word] OR Aerobic Exercise [Text Word] OR Aerobic Exercises [Text Word] OR Exercises, Aerobic [Text Word] OR Exercise Training [Text Word] OR Exercise Trainings [Text Word] OR Training, Exercise [Text Word] OR Trainings, Exercise [Text Word]). We considered all potentially eligible studies for review, irrespective of the primary outcome or language. To identify additional relevant studies, we also conducted a manual search of the reference lists of key articles.

### Study selection and data extraction

Two independent reviewers (HZ and YZ) screened titles and abstracts of the retrieved articles based on the inclusion criteria. In cases where a decision could not be made based on the title and abstract, the full article was read to determine whether the study was eligible. Disagreements between them were resolved by consensus, and a final decision was reached by involving the reviewer (JW). We regarded studies as eligible for inclusion if they were RCTs conducted in adults with any stage of CRC, comparing physical exercise [comprising aerobic exercise (AE), resistance exercise (RE), or their combination] treatment to usual care treatment strategy. Considering that sudden participation in exercise for patients who have been inactive for a long period of time may cause problems such as hypoglycemia, elevated blood pressure, and muscle and joint injuries, and to avoid the influence of such abnormalities on the experimental results, we regarded studies that had at least 4 weeks’ duration of intervention and reported changes in the nutritional status and inflammatory markers of patients with CRC. Exclusion criteria were as follows: observational, retrospective studies, reviews, editorials, animal trials, comments systematic review, and meta-analysis; studies with less than 4 weeks duration of intervention and those that did not indicate exercise type.

The two reviewers (HZ and YZ) used data collection forms presented in Table 1 to independently extract post-intervention outcome variables. Any discrepancies between them were resolved through iteration and discussion. The extracted data were analyzed by two investigators (HZ and FL), while the disagreements were resolved by a third investigator (JN). The extracted data included the following: (1) study characteristics, such as year of publication, sample size, and country; (2) description of the intervention, prescription of exercise program, modality, exercise time per week, frequency, and follow-up duration; (3) participant characteristics, such as age, sex, and CRC stage; (4) outcomes such as levels of BMI, body weight, body fat, waist circumference, level of proinflammatory cytokines IL-6, C-reactive protein (CRP), and TNF-*α*; and (5) secondary outcomes such as quality of life (QoL), fatigue, pain, insomnia, physical functioning, and emotional functioning. All the outcome indicators were presented as the mean ± standard deviation (SD), and for the data in the form of median and interquartile range (IQR), these would be counted after determining whether the data were skewed and then converted by the formula to get the form of mean ± SD (26, 27). Two independent reviewers (YW and FL) assessed the risk of bias according to the PRISMA using the Cochrane Risk of Bias tool. The two reviewers discussed their different views on the risk of bias assessment criteria and tried to reach a consensus through communication and negotiation. The quality of available evidence was assessed using the GRADEpro Guideline Development Tool (GDT).

**Table 1:** Main characteristics of the cohort studies included in the meta-analysis.

Author	Year	Region	*N*	Exercise group			Control group			CRC stage	Intervention						Control	Nutrition indicators	Inflammation marker
				*N*	Meal	age	*N*	Meal	age										
			Summary of all studies								Exercise training								
			996	502	265	59.8 ± 11.5	494	241	59.1 ± 11.5		Type	Frequency	Intensity	supervision status	Time	Duration			
Abrahamson, Page E.	2007	US	184	89	47	55.4 ± 7.0	95	48	55.3 ± 7.4	I-IV	AE, walk	6 d /week	(moderate-to-high)	3 days of supervision and 3 days at home	60 min/d	12 months	Usual care	BMI, Body fat, Glucose, Energy intake	/
AhnAhn, K. Y.	2013	Korea	31	17	12	55.6 ± 7.1	14	5	57.4 ± 6.1	I-III	AE, Walk, Stretching, Core exercise, RE (chest, shoulder, arm, thigh, and calf); balance exercise	7 d /week	(Low-to-moderate)	Supervision twice /day; Unsupervised walk	30 min/d	12 months	Usual care	BMI, Body weight, Fat mass	/
Bousquet-Dion	2018	Canada	63	37	30	73.7 ± 2.5	26	16	69.3 ± 5.1	I-IV	AE, walking, cycling or jogging; +RE, eight exercises targeting major muscle groups of the core, upper, and lower limbs	3–4 d AE/week; 3–4 times RE/week	60–70% VO₂ max (moderate)	Supervision once /week	30 min AE/d; 8–15 Repetitions/ time	8 weeks	Usual care	BMI, Body weight, Fat percentage, Albumin, HbA1C	CRP
Brown, J. C.	2023	US	27	14	7	58.2 ± 9.8	13	4	57.9 ± 9.7	I-III	AE, Running	3–5 d /week	50–70% VO₂ max (moderate)	Unknown	50–60 min/d	24 weeks	Usual care	Body weight, waist circumference	CRP, IL-6, TNF-α
Campbell, K. L.	2008	US	202	100	51	55.3 ± 6.9	102	51	55.1 ± 6.8	I-IV	AE, Running	6 d /week	60–85% VO₂ max (moderate-to-high)	Supervision at least 3 days/week	60 min/d	12 months	Usual care	BMI, Body weight, Height, Body fat	CRP
Carli, Francesco	2020	Canada	110	55	29	78.4 ± 9.3	55	23	80.2 ± 6.9	I-IV	AE, walking; RE, elastic band routine	7 d AE/week; 3 times RE/week	(moderate)	Supervision once/week	30 min AE/d; 30 min RE/ time	4 weeks	Usual care	BMI, Weight, Body fat, Albumin, HbA1C, Hemoglobin	CRP
Devin, J. L.	2019	Australia	20	10	10	66.9 ± 8.4	10	10	64.9 ± 6.0	I- IV	AE, High-intensity interval exercise, Cycling	3 times /week	85–90% VO₂ max Cycling (moderate-to-high)	Unknown	64 min/ time	4 weeks	Usual care	BMI, Body weight	IL-6, TNF-α
Lee, M. K.	2017	Korea	123	62	31	56.3 ± 9.7	61	28	56.3 ± 9.9	II-III	AE, walking, running, hiking;+RE	7 d AE/week; 7d RE/week	65% VO₂ max (moderate)	Home-based exercise and daily text message remind	150 min AE/d; 30 min RE/d	12 weeks	Usual care	BMI, Body weight, Body fat, Waist circumference	CRP, TNF-α
Ligibel, J. A.	2012	US	17	9	5	53.1 ± 10.8	8	4	55. ± 10.6	I-III	AE, walking	7 d /week	(moderate)	Telephone-based supervision once/week	25–50 min/d	16 weeks	Usual care	BMI, Body weight, waist circumference, QoL, fatigue	/
Lin, K. Y.	2014	China	45	21	11	59.0 ± 9.5	24	15	54. ± 10.6	II-III	AE, Cycling, +RE, dumbbell, sandbag, major muscle groups (arms, abdominal muscles, thigh, and gluteus region).	2 times /week	40–75% VO₂ max (moderate)	supervision of the whole process	40–60 min/time	12 weeks	Usual care	BMI, Body weight, Height, Body fat, QoL, fatigue	CRP
Min, J.	2023	Korea	52	26	10	56.6 ± 8.9	26	13	56.4 ± 9.6	I-III	RE, Arm circles, triceps extensions, and posterior pelvic tilt in the supine position; balancing exercises	7 d /week	(low)	Supervision twice/day	30 min /d	4 weeks	Usual care	BMI, Weight, Muscle mass, Body fat, Waist circumference, Thigh circumference	/
Møller, T.	2015	Denmark	31	15	2	57.2 ± 10.3	16	2	47.0 ± 9.2	I- IV	AE, Cycling, ball games, dancing, and cardio Training; RE	3 times /week	(moderate-to-high)	supervision of the whole process	180 min/ time	6/12 weeks	Usual care	BMI, Body weight, QoL, fatigue	/
Toffoli, E. C.	2021	Netherlands	14	8	2	55.1 ± 14.8	6	0	60.7 ± 7.6	II-III	AE, warming-up exercises, training at moderate-high intensity; +RE	2 d /week	(moderate)	supervision of the whole process	30 min AE/d; 20 min RE/d	12 weeks	Usual care	BMI	IL-6
Van Blarigan, E. L.	2022	US	44	22	8	52.2 ± 4.7	22	11	54.1 ± 5.2	I- IV	AE, walking and running	5 d /week	(moderate)	SMS text messages-based supervision	30 min/d	12 weeks	Usual care	BMI, fatigue	/
Van Vulpen, J. K.	2016	Netherlands	33	17	10	58.1 ± 10.3	16	11	58.1 ± 9.6	I- IV	AE, Cycling, +RE, Muscle strength training for arms, legs, shoulders, and trunk	5 d AE/week; 2 times RE/week	Alternating intensity and 45–65% 1-RM RE (moderate)	Suprvision twice/week	40–60 min/time	18 weeks	Usual care	BMI, Body weight, Height, Body fat, Peak VO2, QoL, fatigue	CRP

CRC, colorectal cancer; AE, aerobic exercise; BMI, Body Mass Index; RE, resistance exercise; HbA1C, glycated hemoglobic; CRP, C-reactive protein; IL-6, interleukin-6; TNF-α, tumor necrosis factor-alpha; 1-RM, 1-repetition maximum; QoL, quality of life.

### Statistical analysis

We assessed the effect of exercise for CRC patients on two outcomes: nutrition indicators and inflammation markers. We analyzed these as continuous variables and reported the absolute differences between the arithmetic means before and after the interventions. Stata (version 17.0) and Review Manager 5.4.1, from the Cochrane Collaboration,^2^ were utilized to conduct a quality assessment of the evidence and to calculate effect estimates for combinations of single effects from included studies, as well as to perform subgroup analyses, respectively. The mean differences in the data were calculated at 95% confidence intervals. We used the Cochran Q test to assess heterogeneity between studies. We also did I^2^ testing to assess the magnitude of the heterogeneity between studies, with values greater than 50% regarded as being indicative of moderate-to-high heterogeneity. The results were summarized using random effects models, taking into account the potentially large heterogeneity of the population of interest as well as the details of physical exercise with respect to the clinical methodology. We only conducted descriptive analysis for studies from which the data format could not be obtained. Sensitivity analysis was performed by assessing the impact of individual studies on the overall effect size and one-by-one elimination method to explore sources of heterogeneity, followed by subgroup analyses for intervention duration, exercise types, exercise time, and patient BMIs. Datasets from inflammation markers were presented as forest plots, and the subgroup analyses were presented in statistical tables. We assessed the possibility of publication bias by constructing a funnel plot of each trial’s effect size against the standard error when the comparison included more than three studies. We assessed funnel plot asymmetry using Begg and Egger tests and defined significant publication bias as a *p*-value of <0.05.

### Role of the funding source

The study was supported by intramural funds, with no commercial entity involved. The funding source had no role in study design, data collection, data analysis, data interpretation, or writing of the report. The corresponding author had full access to all the data in the study and had final responsibility for the decision to submit for publication.

## Results

### Search results and study characteristics

**Figure 1:**
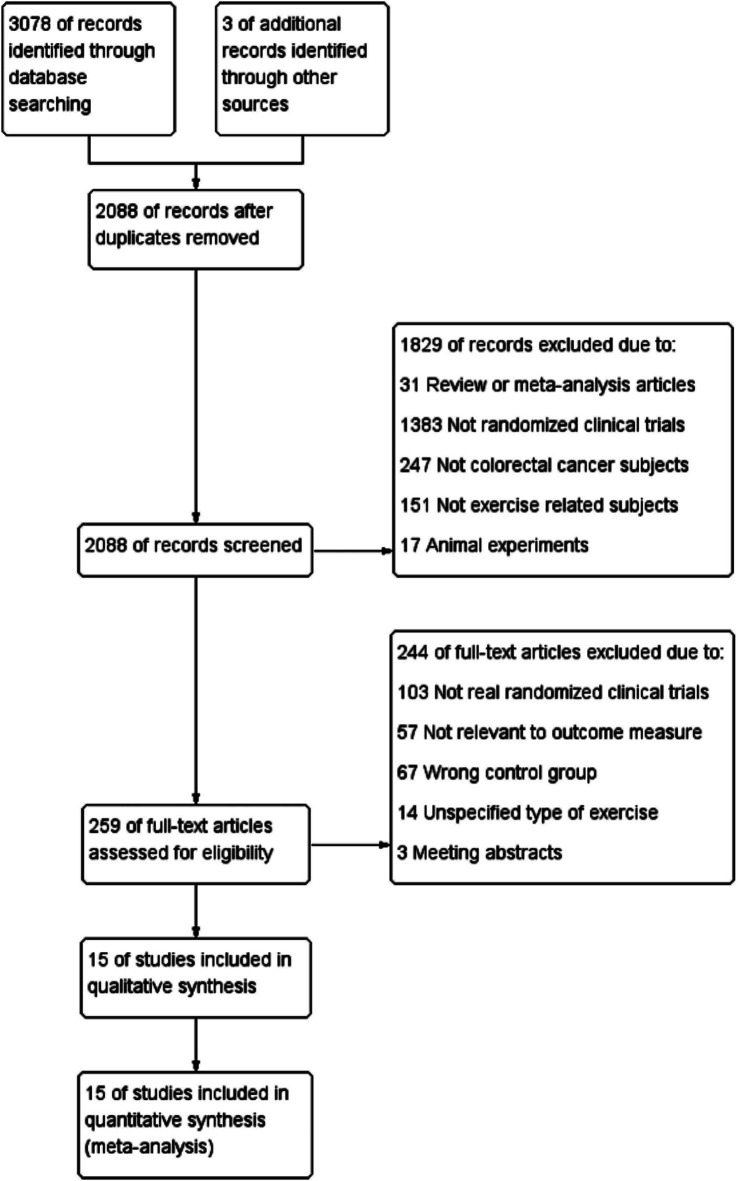
Flow diagram of the trial selection process.

The search strategy identified 3,081 articles. Among them, 15 studies (28–42), comprising 996 patients, met the inclusion criteria and were therefore included in the final analysis (Figure 1). All 15 trials were published between 2007 and 2023, and the main characteristics of the included articles are outlined in Table 1. The mean age of patients was 59.45 ± 11.48 years, the mean duration was 18.8 weeks, and the mean baseline BMI was 26.88 ± 4.77 kg/m^2^. In summary, patients had stages 1–3 and 1–4 CRC; Six studies reported an intervention duration of >12 weeks (28, 29, 31, 32, 36, 42), while nine studies reported ≤12 weeks (30, 33–35, 37–41). In this meta-analysis, interventions lasting >12 weeks were categorized as ‘long-term exercise’, as they typically allow for physiological adaptation across systems (cardiorespiratory, metabolic, etc.) (13, 43, 44). In terms of the type of exercise training program, six articles described AE (28, 31, 32, 34, 36, 41), whereas seven were combined AE with RE (28, 33, 35, 37, 39, 40, 42). Exercise time was less than 300 min per week in nine studies (29–31, 34, 37, 38, 40–42) and more than 300 min per week in six studies (28, 32, 33, 35, 36, 39). There were four studies (28, 32, 34, 39) with exercise intensity designed at moderate-high intensity and 11 (29–31, 33, 35–38, 40–42) set at low-moderate intensity. Regarding the exercise supervision status, except for Brown, J. C. (2023) (31) and Devin, J. L. (34), the supervision status was not explicitly elucidated; the rest of the RCT studies were completed under the supervision of exercise physiologists, with a completion rate of >80%. Thus, we considered that exercise supervision had minimal effect on the heterogeneity of the experimental results (45).

### Quality assessment

**Figure 2:**
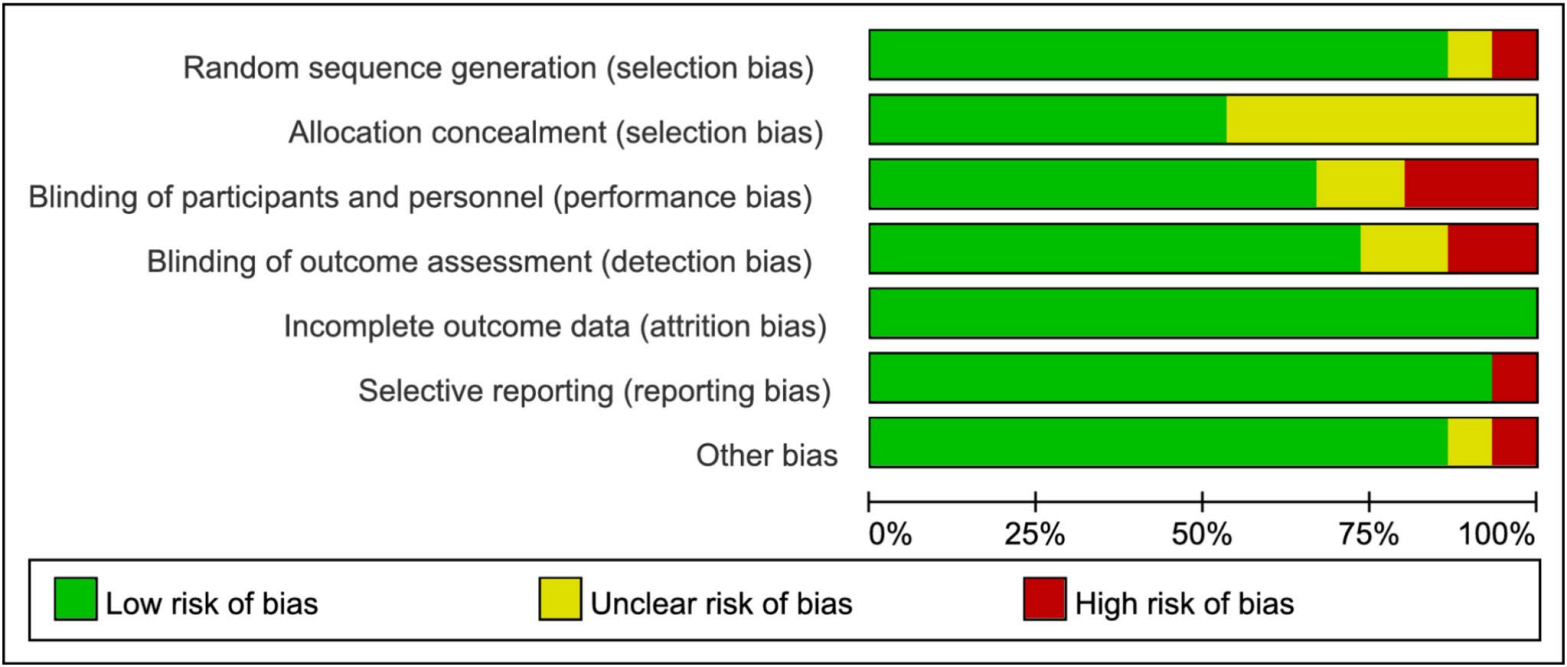
Risk of bias graph.

Figure 2 and Supplementary Figure S1 depict the risk of bias assessment for the trials. All studies were randomized controlled trials (RCTs), and none were stopped early. Four studies reported methods using computer-generated random numbers for randomization (28, 35, 39, 42). One trial described allocation based on participant preference (“The participants in this 2-group controlled trial were allocated to either the supervised-exercise group or the usual-care group on the basis of their preference after inclusion and baseline testing”) (37), which may introduce selection bias and compromise the validity of randomization. Therefore, this approach raised concerns about potential bias in the assignment process. Four RCTs had a high risk of performance bias due to insufficient blinding of investigators or patients (29, 31, 38, 42), and two RCTs lacked blinding during data analysis (34, 39). Eight studies had a low risk of reporting bias due to systematic clinical trial registration (31, 33, 35, 38–42). One study had incomplete or inaccessible reporting of some outcome indicators (31), and another study may have had baseline imbalance in data such as age (41). All included studies exhibited low publication bias. The quality of evidence for BMI, body weight, and body fat was assessed as high, supported by adequate sample sizes and a low risk of bias. Conversely, outcomes such as waist circumference, CRP, IL-6, TNF-*α*, QoL (quality of life), fatigue, pain, insomnia, physical functioning, and emotional functioning were rated as moderate quality due to small sample sizes (Table 2).

**Table 2:** Quality assessment of outcome (GRADE).

Certainty assessment							No of patients		Effect		Certainty	Importance
№ of studies	Study design	Risk of bias	Inconsistency	Indirectness	Imprecision	Other considerations	Usual care	Exercise training	Relative (95% CI)	Absolute (95% CI)		
BMI												
14	randomized trials	not serious	not serious	not serious	not serious	none	488	481	–	MD 0.38 lower (0.92 lower to 0.15 higher)	⨁⨁⨁⨁ High	CRITICAL 7
Body weight												
11	randomized trials	not serious	not serious	not serious	not serious	none	370	356	–	MD 0.81 lower (3.04 lower to 1.42 higher)	⨁⨁⨁⨁ High	CRITICAL 7
Body fat												
7	randomized trials	not serious	serious	not serious	not serious	none	386	379	–	MD 1.36 lower (2.11 lower to 0.61 lower)	⨁⨁⨁ High	IMPORTANT 6
waist circumference												
3	randomized trials	not serious	not serious	not serious	not serious	none	97	95	–	MD 0.09 lower (2.67 lower to 2.49 higher)	⨁⨁⨁ Moderate	IMPORTANT 5
CRP												
4	randomized trials	not serious	not serious	not serious	not serious	none	213	202	–	MD 0.33 lower (0.62 lower to 0.04 lower)	⨁⨁⨁ Moderate	IMPORTANT 5
IL-6												
3	randomized trials	not serious	serious	not serious	not serious	none	32	29	–	MD 0.14 higher (0.13 lower to 0.41 higher)	⨁⨁◯ Moderate	IMPORTANT 5
TNF-α												
3	randomized trials	not serious	serious	not serious	not serious	none	86	84	–	MD 0.28 higher (0.21 lower to 0.77 higher)	⨁⨁◯ Moderate	IMPORTANT 5
QoL												
3	randomized trials	not serious	not serious	not serious	not serious	none	41	40	–	MD 0.33 higher (5.54 lower to 6.20 higher)	⨁⨁◯ Moderate	IMPORTANT 5
Fatigue												
4	randomized trials	not serious	not serious	not serious	not serious	none	62	64	–	MD 2.39 lower (7.46 lower to 2.68 higher)	⨁⨁◯ Moderate	IMPORTANT 5
Pain												
4	randomized trials	not serious	not serious	not serious	not serious	none	62	64	–	MD 2.45 lower (11.51 lower to 6.61 higher)	⨁⨁◯ Moderate	IMPORTANT 5
Insomnia												
3	randomized trials	not serious	not serious	not serious	not serious	none	47	48	–	MD 4.42 lower (14.89 lower to 6.06 higher)	⨁⨁◯ Moderate	IMPORTANT 5
Physical functioning												
3	randomized trials	not serious	not serious	not serious	not serious	none	53	56	–	MD 6.51 higher (0.41 higher to 12.61 higher)	⨁⨁◯ Moderate	IMPORTANT 5
Emotional functioning												
3	randomized trials	not serious	not serious	not serious	not serious	none	53	56	–	MD 10.1 higher (2.02 higher to 18.18 higher)	⨁⨁◯ Moderate	IMPORTANT 5

CI, confidence interval; MD, mean difference; BMI, Body Mass Index; CRP, C-reactive protein; IL-6, interleukin-6; TNF-α, tumor necrosis factor-alpha; QoL, Quality of life.

^a^High heterogeneity (*I*^2^ > 50%). ⨁◯Indicates rating of quality of evidence.

### Effects on nutrition indicators

**Figure 3:**
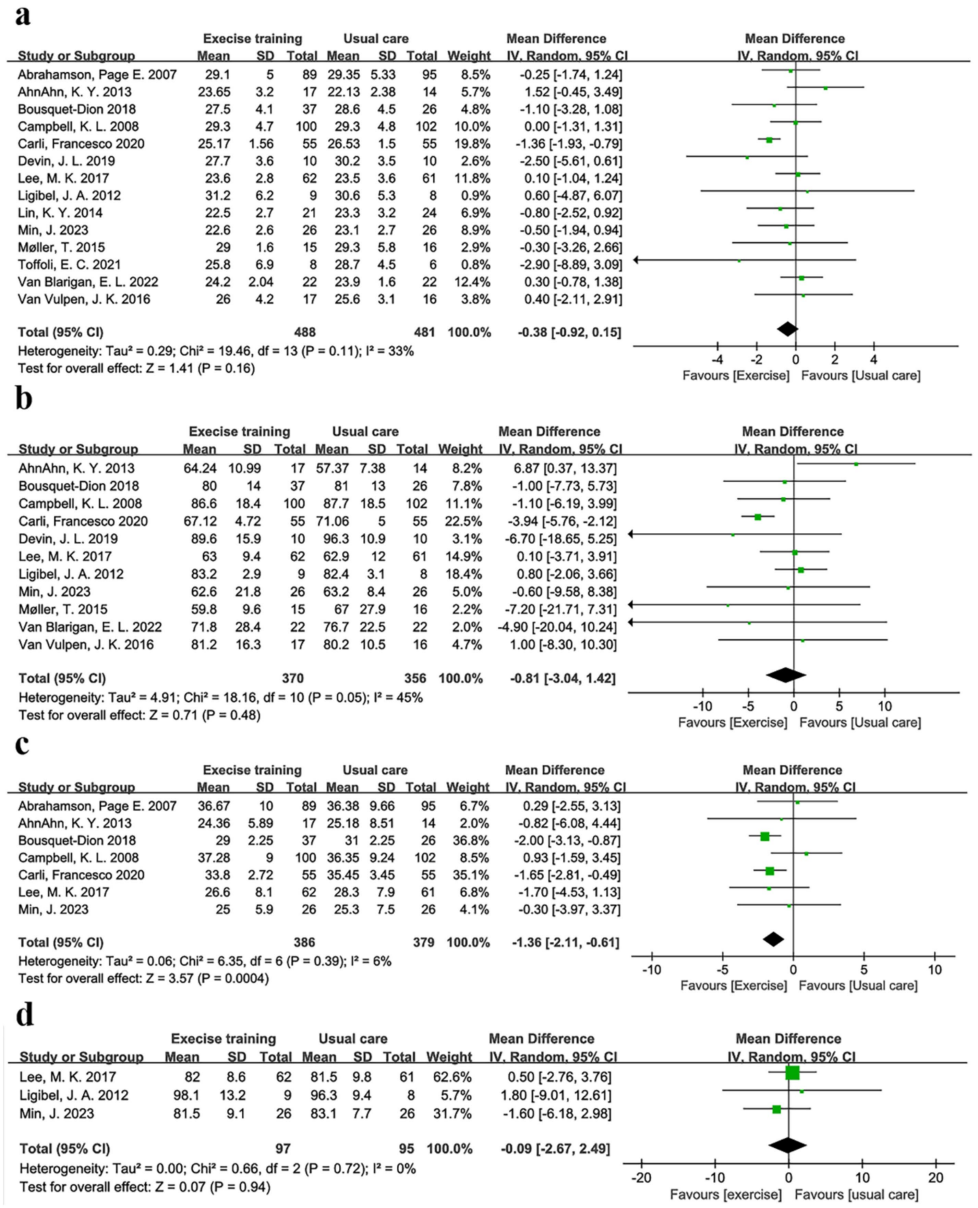
Forest plot of the physical exercise vs. usual care on the CRC patients’ indicators of nutrition, **(a)** BMI (kg/m^2^); **(b)** Body weight (kg); **(c)** Body fat (%); and **(d)** Waist circumference (cm).

In a pooled analysis of all 15 trials, physical exercise improved nutritional indicators (BMI, weight, and body fat) in CRC patients when compared to usual care. Fourteen studies (28–30, 32–42), comprising 488 exercise and 481 usual care participants, reported BMI. Figure 3a and Table 3 showed no significant between-group difference in BMI (*p* = 0.16), with low heterogeneity (I^2^ = 33%). Supplementary Figure S2a demonstrated a symmetric funnel plot, and both Begg’s test (*p* = 0.443) and Egger’s test (*p* = 0.146) indicated no significant publication bias. Sensitivity analysis (Supplementary Figure S3a) showed robust results for BMI. Subgroup analyses of exercise duration, volume, intensity, baseline BMI, and type (shown in Supplementary Figure S4) revealed that trials with duration ≤12 weeks showed a BMI reduction of 0.38 kg/m^2^ (95% CI -1.15 to −0.02, *p* = 0.04, I^2^ = 33%), while those with >12 weeks showed no significant change (*p* = 0.45). Combined AE + RE reduced BMI by 0.85 kg/m^2^ (95% CI -1.45 to −0.25, *p* = 0.005, I^2^ = 14%), whereas aerobic-only exercise had no effect (*p* = 0.90). No significant differences were observed for exercise volume (< vs. ≥300 min/week, *p* = 0.17–0.60), baseline BMI (overweight vs. non-overweight, *p* = 0.07–0.97), or intensity (low-moderate vs. moderate-high, *p* = 0.34–0.48) (Figure 3; Table 3).

**Table 3:** The effects of physics exercise on CRC patients.

Outcome		Trials	Number		Meta-analysis of changes					
			Exercise	Usual care	Est (95% CI)	*Z*	*p*	I^2^ (%)	Begg	Egger
BMI (kg/m^2^)		14	488	481	−0.38[−0.92, 0.15]	1.41	0.16	33	0.443	0.416
Exercise duration	Duration ≤ 12 weeks	10	356	348	−0.59[−1.15, −0.02]	2.02	0.04	33	/	/
	Duration>12 weeks	4	132	133	0.40 [−0.65, 1.46]	0.75	0.45	0	/	/
Exercise time	Time<300 min/week	8	158	144	−0.2[−0.92, 0.53]	0.53	0.60	14	/	/
	Time ≥ 300 min/week	6	330	337	−0.52[−1.25, 0.22]	1.38	0.17	40	/	/
Exercise type	AE	5	230	237	−0.05[−0.75, 0.56]	0.13	0.9	0	/	/
	AE + RE	7	215	204	−0.85[−1.45, −0.25]	2.78	0.005	14	/	/
Patient’s BMI	BMI ≥ 25 kg/m^2^	10	362	356	−0.58[−1.21, 0.05]	1.81	0.07	28	/	/
	BMI<25 kg/m^2^	4	126	125	−0.01[−0.84, 0.81]	0.04	0.97	17	/	/
Intensity	Moderate-high	4	214	223	−0.32[−1.22, 0.57]	0.71	0.48	0	/	/
	Low- moderate	10	274	258	−0.34[−1.03, 0.35]	0.96	0.34	47	/	/
Body weight (kg)		11	370	356	−0.81[−3.04, 1.42]	0.71	0.48	45	0.755	0.910
Exercise duration	Duration ≤ 12 weeks	7	227	216	−3.12[−4.66, −1.58]	3.98	<0.0001	0	/	/
	Duration>12 weeks	4	143	140	1.34 [−1.48, 4.15]	0.93	0.35	21	/	/
Exercise time	Time<300 min/week	6	129	114	0.73 [−3.12, 4.58]	0.37	0.71	13	/	/
	Time ≥ 300 min/week	5	208	209	0.03 [−2.02, 2.07]	0.03	0.98	0	/	/
Exercise type	AE	4	141	142	−0.08 [−2.49, 2.33]	0.06	0.95	0	/	/
	AE + RE	5	186	174	−2.45[−4.68, −0.21]	2.14	0.03	19	/	/
Patient’s BMI	BMI ≥ 25 kg/m^2^	8	265	255	−1.95 [−4.08, 0.18]	1.79	0.07	26	/	/
	BMI<25 kg/m^2^	3	105	101	2.00 [−2.51, 6.51]	0.87	0.39	40	/	/
Intensity	Moderate-high	3	125	128	−2.45[−6.91, 2.00]	1.08	0.28	0	/	/
	Low- moderate	8	245	22	−0.26[−2.97, 2.45]	0.19	0.85	59	/	/
Body fat (%)		7	386	379	−1.36[−2.11, −0.61]	3.57	0.0004	6	0.368	0.101
exercise duration	Duration ≤ 12 weeks	4	180	168	−1.75[−2.52, −0.99]	4.52	<0.0001	0	/	/
	Duration>12 weeks	3	206	211	0.48[−1.29, 2.26]	0.53	0.59	0	/	/
Exercise time	Time<300 min/week	3	80	66	−1.81[−2.87, −0.75]	3.36	0.0008	0	/	/
	Time ≥ 300 min/week	4	306	313	−0.82[−2.14, 0.49]	1.23	0.22	33	/	/
Patient’s BMI	BMI ≥ 25 kg/m^2^	4	281	278	−1.12[−2.3, 0.05]	1.88	0.06	49	/	/
	BMI<25 kg/m^2^	3	105	101	−1.12[−3.18, 0.94]	1.07	0.29	0	/	/
Intensity	Moderate-high	2	189	197	0.65[−1.24, 2.53]	0.68	0.50	0	/	/
	Low- moderate	5	197	182	−1.74[−2.49,-0.98]	4.52	<0.0001	0	/	/
Waist circumference (cm)		3	97	95	−0.09[−2.67, 2.49]	0.07	0.94	0	1	0.938
QoL		3	41	40	0.33[−5.54, 6.2]	0.11	0.91	0	1	0.693
Fatigue		4	62	64	−2.39[−7.46, 2.68]	0.92	0.36	7	1	0.915
Exercise time	Time≥300 min/w	3	41	40	0.33[−5.54, 6.20]	0.11	0.91	0	/	/
Exercise type	AE + RE	3	53	56	−5.73[−12.21, 0.76]	1.73	0.08	0	/	/
Intensity	Low- moderate	3	47	48	−2.93[−9.31, 3.44]	0.9	0.37	35	/	/
Pain		4	62	64	−2.45[−11.51, 6.61]	0.53	0.60	34	0.734	0.160
Exercise time	Time≥300 min/w	3	41	40	3.91[−6.58, 14.39]	0.73	0.47	0	/	/
Exercise type	AE + RE	3	53	56	−1.52[−13.31, 0.26]	0.25	0.8	56	/	/
Intensity	Low- moderate	3	47	48	−4.67[−14.68, 5.33]	0.92	0.36	31	/	/
Insomnia		3	47	48	−4.42[−14.89, 6.06]	0.83	0.41	0	1	0.952
Physical functioning		3	53	56	6.51[0.41, 12.61]	2.09	0.04	0	0.296	0.419
Emotional functioning		3	53	56	10.1[2.02, 18.18]	2.45	0.01	41	1	0.136

CRC, colorectal cancer; BMI, Body Mass Index; AE, aerobic exercise; RE, resistance exercise; QoL, Quality of life.

Body weight was another key nutritional indicator. Eleven studies comprising 370 exercise and 356 usual care participants reported body weight (29, 30, 32–36, 38, 39, 41, 42). As Table 3 and Figure 3b show, there was no significant between-group difference in body weight (*p* = 0.48), with low heterogeneity (I^2^ = 45%). Supplementary Figure S2b showed a symmetric funnel plot, and both Begg’s test (*p* = 0.755) and Egger’s test (*p* = 0.910) indicated no publication bias. Sensitivity analysis (Supplementary Figure S3b) confirmed stable results for body weight. Subgroup analyses revealed that trials with duration ≤12 weeks showed a body weight reduction of −3.12 kg (95% CI -4.66 to −1.58, *p* < 0.0001, I^2^ = 0%). In addition, the AE + RE group had a body weight reduction of −2.14 kg (95% CI -4.68 to −0.21, p < 0.0001, I^2^ = 0%), while no significant changes were observed in the >12 weeks group (*p* = 0.35) or AE-only group (*p* = 0.95). In addition, no significant differences were found for exercise volume, intensity, or baseline BMI (Supplementary Figure S5; Table 3). These findings suggest that physical exercise is effective across varying baseline BMI levels, with short-duration AE + RE combinations particularly helpful for improving BMI and body weight.

Body fat, defined as the weight of body fat as a percentage of total body weight, provides a more accurate measure of obesity than BMI (46). Body fat was reported in seven trials (28–30, 32, 35, 38), comprising 386 exercise and 379 usual care participants. Table 3 and Figure 3c showed that body fat decreased by −1.36% (95% CI -2.11 to −0.61, *p* = 0.0004, I^2^ = 6%) in the exercise group compared to the usual care group. Supplementary Figure S2c showed a symmetric funnel plot, with both Begg’s test (*p* = 0.368) and Egger’s test (*p* = 0.10) indicating no significant publication bias. Sensitivity analysis (Supplementary Figure S3c) confirmed the stability of body fat results. Notably, body fat was reduced by −1.75% (95% CI -2.52 to −0.99, *p* < 0.0001, I^2^ = 0%) in the ≤12 weeks group, by −1.81% (95% CI -2.87 to −0.75, *p* = 0.0008, I^2^ = 0%) in the <300 min/week group, and by −1.74% (95% CI -2.49 to −0.98, p < 0.0001, I^2^ = 0%) in the low-moderate intensity group. However, no significant changes were observed in the BMI < 25 kg/m^2^ group (*p* = 0.29), BMI ≥ 25 kg/m^2^ group (*p* = 0.06), or moderate-high intensity group (*p* = 0.50). Waist circumference was measured in three studies, including 193 patients (35, 36, 38). Meta-analysis showed no significant effect of physical exercise on waist circumference compared to usual care (*p* = 0.94, *I*^2^ = 0%) (Figure 3d), with Begg’s test (*p* = 1) and Egger’s test (*p* = 0.938) indicating no publication bias (Supplementary Figure S6; Table 3).

### Effects on inflammation markers

**Figure 4:**
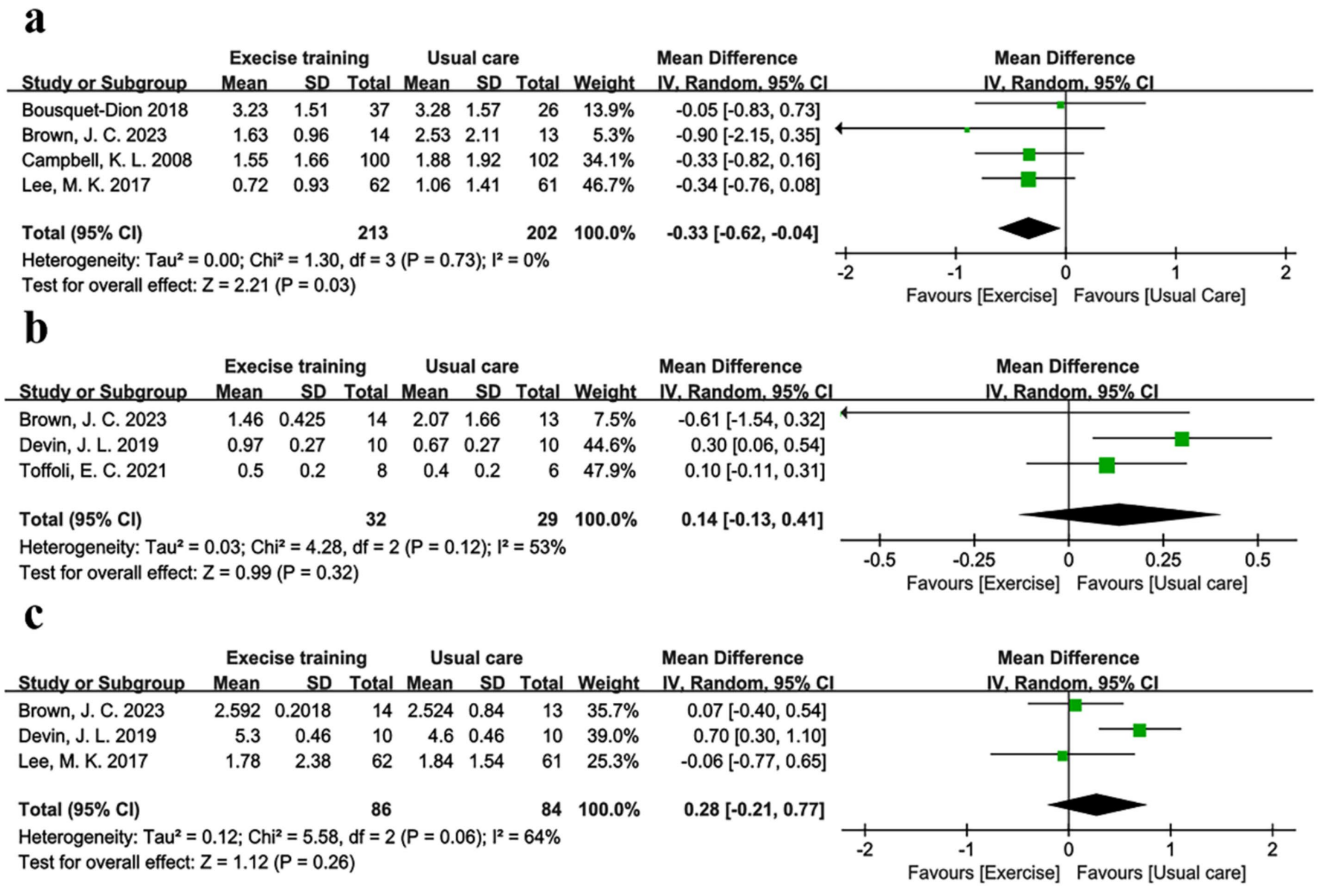
Forest plot of the physical exercise vs. usual care on the CRC patients’ inflammation markers, **(a)** CRP (mg/dL); **(b)** IL-6 (pg/mL) and **(c)** TNF-*α* (pg/mL).

Evidence supporting the role of inflammation in colorectal carcinogenesis is growing (47). For example, inflammatory bowel disease, characterized by local colon inflammation, is associated with increased colorectal cancer risk (48). Chronic inflammation may initiate and promote carcinogenesis through proinflammatory cytokines (e.g., IL-6) and reactive oxygen species, which activate transcription factors driving tumor growth (49, 50). Meta-analysis results showed that physical exercise improved inflammatory profiles. Four studies (30–32, 35), including 213 exercise and 202 usual care participants, reported C-reactive protein (CRP). Figure 4a showed a significant CRP reduction of −0.33 mg/dL (95% CI -0.62 to −0.04, *p* = 0.03, I^2^ = 0%) in the exercise group. Supplementary Figure S2d showed a symmetric funnel plot, with Begg’s (*p* = 1.0) and Egger’s (*p* = 0.653) tests indicating no publication bias. IL-6 was reported in three studies with marginal non-significance (*p* = 0.060, I^2^ = 0%), but no significant group differences were observed in IL-6 (31, 34, 40) (3 studies, 71 participants, *p* = 0.32, I^2^ = 53%) or TNF-*α* (31, 34, 35) (3 studies, 170 participants, *p* = 0.26, I^2^ = 64%) (Figures 4b,c). High heterogeneity (I^2^ > 50%) in IL-6 and TNF-α analyses prompted the use of the random-effects model and leave-one-out sensitivity analyses. Excluding Brown, J. C. (31), reduced IL-6 heterogeneity (I^2^ from 53 to 34%), while excluding Devin et al. (34), eliminated TNF-α heterogeneity (I^2^ from 64 to 0%), indicating these studies were primary heterogeneity sources. Notably, Brown, J. C. (31), did not report exercise supervision, which may have affected adherence, while Devin, J. L. (34), used high-intensity exercise, potentially influencing TNF-α dynamics. Despite high heterogeneity, publication bias was absent for both markers (IL-6: Begg’s *p* = 1, Egger’s *p* = 0.473; TNF-α: Begg’s p = 1, Egger’s *p* = 0.462).

### Effects on secondary outcomes

Exercise-induced improvements in QoL were multifaceted. It has been shown that physical exercise can alleviate symptoms such as fatigue, pain, and insomnia, which cancer patients commonly experience. It can also enhance psychological wellbeing by reducing anxiety and depression, fostering a sense of control and empowerment, and improving social interactions and support. These psychosocial benefits are crucial, as mental health significantly influences overall health outcomes and QoL in cancer patients (51, 52). Therefore, this study examined the secondary outcomes (e.g., QoL, fatigue, pain, etc.) of exercise in CRC patients. A total of three studies (36, 39, 42), comprising 41 participants who received physical exercise and 40 usual care subjects, reported QoL. Supplementary Figure S7 and Table 3 showed no significant benefit (*p* = 0.91, *I*^2^ = 0%) for QoL in the exercise group. In addition, some signature indicators in the EORTC QLQ-C30 form were statistically analyzed (53). Physical functioning and emotional functioning were reported in three trials (37, 39, 42), comprising 53 participants who received physical exercise and 56 usual care subjects. The meta-analysis showed that physical functioning increased by 6.51 (95% CI 0.41 to 12.61, *p* = 0.04, *I*^2^ = 0) in the physical exercise group compared with the usual care group, while emotional functioning increased by 10.1 (95% CI 2.02 to 18.18, *p* = 0.01, *I*^2^ = 41%), as Table 3 shows. Moreover, there was no significant benefit for fatigue (*p* = 0.36, *I*^2^ = 7%), pain (*p* = 0.60, *I*^2^ = 34%), and insomnia (*p* = 0.41, *I*^2^ = 0) in the exercise group. Furthermore, subgroup analyses showed no significant differences in fatigue and pain indicators across different exercise durations, types, and intensities. All secondary outcomes showed *I*^2^ values <50%, indicating low heterogeneity. Begg’s test and Egger’s test values were >0.05, which indicate no significant publication bias.

## Discussion

Our meta-analysis results show that, compared with usual care treatments, additional physical exercise can yield reduced inflammation and improve nutritional status and physical and emotional functioning. Compared with usual care, additional physical exercise was effective in lowering body fat (−1.36%), reducing CRP concentrations (−0.33 mg/dL), and improving physical functioning (+6.51) and emotional functioning (+10.1). Furthermore, no significant differences were found in waist circumference, IL-6, TNF-*α*, QoL, fatigue, pain, and insomnia between patients who underwent physical exercise programs relative to usual care. Subgroup analyses further revealed that short-duration exercise (≤12 weeks) significantly reduced patients’ BMI, body weight, and body fat. Additionally, exercise with low-moderate intensity and short time (<300 min/week) was associated with reduced body fat, while the combination of AE and RE effectively lowered BMI and body weight. However, no significant differences were observed in subgroups of long duration, high-volume, aerobic-only, or moderate-to-high intensity exercise, nor in subgroups stratified by pre-intervention BMI. These findings highlight the optimal exercise parameters for improving metabolic and functional outcomes in CRC patients. Therefore, these data support the use of additional physical exercise as a primary care strategy for CRC patients.

As a cancer of the bowel, CRC is primarily characterized by malignant transformations of colonic or rectal epithelial cells, with multifactorial origins including genetic predisposition, environmental exposures, and dietary patterns. Since the colorectum is the last organ in the body that digests food, it is responsible for processing large amounts of waste and toxins. Prolonged retention of intestinal waste may contribute to mucosal irritation and increase the risk of colorectal carcinogenesis, although the exact mechanistic link remains under investigation. Obesity and chronic inflammation, as two of the main reasons for the high incidence of colon cancer, should be avoided even after colon cancer surgery (54). In addition, obese people are often in a state of chronic inflammation, which might induce various vascular diseases (55, 56). Postoperative obesity would have a detrimental impact on the recovery of CRC patients. First of all, obesity would lead to hormonal imbalance in the body, which will affect the microbial communities in the intestines. These microbial communities played an important role in the health of the gut, which was responsible for digesting food, absorbing nutrients, and eliminating waste and toxins. It might lead to gut inflammation and a host of other health problems when the microbiome is out of balance (57). In addition, obesity might cause inflammatory responses in the body, which was detrimental for the recovery and repair of the gut (58, 59). If the bowel were not restored and repaired in a timely manner, it would have a significant negative impact on the recovery of CRC patients. Previous studies have shown that BMI was incrementally associated with wound-related complications, illustrating how the proliferation of obesity relates to a growing risk for surgical complications (60). Moreover, laparoscopic colorectal cancer operations in obese patients pose an increased technical challenge, as demonstrated by higher conversion rates and higher risk of postoperative complications compared to non-obese patients (61). Therefore, it was needed for CRC patients to avoid obesity after surgery. Specifically, CRC patients should follow a healthy diet and lifestyle, control their body weight, and maintain moderate exercise and sleep quality.

CRP, as the most widely used and sensitive marker for determining the inflammatory condition of patients, was an important reference for the diagnosis and treatment of infectious diseases. In addition, previous studies have shown that CRP has been identified as a potential prognostic indicator in CRC, reflecting systemic inflammation associated with tumor progression (62). Elevated levels of CRP enable the formation of a tumor microenvironment that is conducive to sustained tumor growth, invasion, and metastatic conditions (63, 64). Systemic inflammatory response is strongly linked to cancer development, progression, and poor prognosis. There were studies that investigated the effect of CRP levels on the prognosis of CRC patients, and the experimental results showed a linear relationship between CRP levels and poor postoperative prognosis of CRC patients (65, 66). Exercise to reduce the inflammatory profile of patients was actually well understood, as a recent study by Justin C. Brown et al. (9) showed that physical exercise lowered CRP in CRC patients by nearly 35% and that this trend was linear with the rate of exercise attainment. There are positive implications for patient prognosis and recovery in terms of reducing the patient’s inflammatory profile. In addition to antibiotic anti-inflammatory drugs and non-steroidal anti-inflammatory drugs, recent studies have shown that saponins can suppress intestinal inflammation, promote intestinal barrier repair, maintain the diversity of the intestinal flora, and decrease the incidence rate of colon-inflammation-related colon cancer (67). Among the various non-pharmacological strategies that have been investigated, different modalities of exercise training, such as endurance, resistance training, and combined training, appear to act favorably in controlling inflammation, as they are capable of inducing an increase in anti-inflammatory cytokine secretion by adipose tissue causing significant reduction in CPR levels (68). These approaches are similar in nature, all aiming to alleviate inflammation by restoring intestinal flora.

Findings from our meta-analysis show an overall beneficial effect on additional physical exercise in CRC patients. As shown in Figure 4 and Table 3, our trial has yielded robust and consistent findings that physical exercise improved obesity in CRC patients, with BMI, weight, and body fat all decreasing compared to usual care. The reduction of obesity indirectly improved CRP levels and reduced inflammation in patients. It is worth mentioning that this trial also elaborates on the impact of exercise modalities and intensity on CRC patients. Since strenuous postoperative exercise might increase the risk of wound tearing and infection in patients, an appropriate training duration (<300 min/week) and low-to-moderate exercise intensity seems to be more appropriate. A questionnaire on movement barriers for colon cancer patients showed that common movement barriers included treatment-related effects and lack of time (69, 70). In the short term, low-to-moderate intensity supervised physical exercise was more appropriate for patient safety after surgery. Therefore, there will be relatively fewer obstacles in the clinical implementation phase. In addition, a recent study by Justin Y. Jeon et al. had shown that a similar exercise program reduces the length of stay in the hospital and duration of flatulence in CRC patients after surgery (71). In addition, AE could help recovery and avoid bowel adhesion, and RE might help improve inflammation. Therefore, the combination of AE and RE is more suitable for CRC patients’ recovery. The observed improvements in physical/emotional functioning may be mediated by mechanisms such as reduced inflammation, immune modulation, and hormonal regulation, although these did not translate to significant global QoL improvements in our analysis. This may be attributed to the multidimensional nature of QoL evaluation, which encompasses not only physical and emotional functions but also dimensions such as social function and symptom burden (e.g., fatigue, pain, and insomnia). Notably, these latter dimensions showed no significant improvement with exercise intervention in our study (*p* > 0.30 for all), potentially offsetting the positive effects of enhanced physical and emotional functioning on global QoL. Studies have shown that exercise reduces pro-inflammatory cytokines and increases anti-inflammatory cytokines, thereby reducing chronic inflammation associated with cancer progression and treatment side effects. In addition, exercise can help regulate hormones such as insulin, cortisol, and dopamine to improve overall metabolic fitness and fatigue, as well as improve emotional functioning such as motivation and attention (72, 73). Beyond physical benefits, exercise may offer psychological advantages by promoting a sense of agency and emotional regulation, akin to mind–body therapies (52, 72). Indeed, this consistency was apparent even though these studies differed in several ways, including the country of trial patients, central base or home base exercise venue, exercise intensity, and exercise program. Taken together, these studies are supportive of the generalizability across clinical settings of the observed beneficial effects of additional physical exercise nursing strategy.

### Study limitations

A limitation of this analysis is that the long-term durability of this treatment is unknown; included trials ranged in duration from 4 weeks to 12 months (mean 18.8 weeks). Second, although most of the included studies were published in high-impact journals, there were study features that carry a potential risk of bias, such as the fact that only eight studies published clinical trial registration. In addition, most of the trials included in this meta-analysis were based on small sample sizes, except for three outcome indicators, namely BMI, body weight, and body fat. Meta-analyses with small sample sizes are prone to bias in tests of heterogeneity based on chi-square tests (e.g., the I^2^ statistic); for instance, in sensitivity analyses of IL-6/TNF-*α*, the stability of study-specific effects and meta-regression results was compromised due to insufficient data, potentially leading to false conclusions about heterogeneity (e.g., underestimating true variability). In addition, the small sample size limited the feasibility of subgroup analyses of data on pain and fatigue in this study. Furthermore, the lack of standalone RE trials (only two studies) prevented subgroup analysis of RE-specific effects, possibly due to unpublished negative results. Last but not least, many of the outcome metrics reported in fewer studies could not be analyzed, such as skeletal muscle mass, glycated hemoglobin A1c (HbA1c), IGF-1, and other inflammation markers such as serum amyloid A (SAA) and procalcitonin (PCT), which hinders understanding of exercise’s biological pathways in CRC. Therefore, further large-scale (≥200 participants per arm), multi-center studies with longer exercise interventions and more sensitive indicators are needed to validate the effects of physical exercise on CRC patients.

## Conclusion

This meta-analysis indicates that physical exercise may improve obesity and reduce inflammation in CRC patients. In addition, exercise modalities that combine short-term, moderate-volume exercises and AR + RE are more beneficial. Although further studies and high-quality evidence are needed to establish the optimal approach to the application of this treatment in practice, our findings lend support to physical exercise as a potential adjuvant nursing intervention.

## Footnotes

^1^ https://clinicaltrials.gov/

^2^ www.cochrane.org
