# Supplementary material for: Physical exercise as a potential adjuvant therapy: effects on inflammation and nutrition in colorectal cancer patients—a systematic review and meta-analysis
Source: Front Nutr. 2025 Jun 26;12:1612674. doi: 10.3389/fnut.2025.1612674 (PMC12243031; doi:10.3389/fnut.2025.1612674)
Supplement: Supplementary file 1 [file Table_1.docx]

Table.S1 search strategy

| **Databases** | **Search strategy** |
| --- | --- |
| Pubmed central | (“Colonic Neoplasms” [MeSH Terms] OR Colonic Neoplasm [Text Word] OR Neoplasm, Colonic [Text Word] OR Neoplasms, Colonic [Text Word] OR Colon Neoplasms [Text Word] OR Colon Neoplasm [Text Word] OR Neoplasm, Colon [Text Word] OR Neoplasms, Colon [Text Word] OR Cancer of Colon [Text Word] OR Colon Cancers [Text Word] OR Colon Cancer [Text Word] OR Cancer, Colon [Text Word] OR Cancers, Colon [Text Word] OR Cancer of the Colon [Text Word] OR Colonic Cancer [Text Word] OR Cancer, Colonic [Text Word] OR Cancers, Colonic [Text Word] OR Colonic Cancers [Text Word] OR Colon Adenocarcinoma [Text Word] OR Adenocarcinoma, Colon [Text Word] OR Adenocarcinomas, Colon [Text Word] OR Colon Adenocarcinomas [Text Word] OR CRC [Text Word]) AND (“Exercise” [MeSH Terms] OR Exercises [Text Word] OR Physical Activity [Text Word] OR Activities, Physical [Text Word] OR Activity, Physical [Text Word] OR Physical Activities [Text Word] OR Exercise, Physical [Text Word] OR Exercises, Physical [Text Word] OR Physical Exercise [Text Word] OR Physical Exercises [Text Word] OR Acute Exercise [Text Word] OR Acute Exercise [Text Word] OR Exercise, Acute [Text Word] OR Exercises, Acute [Text Word] OR Exercise, Isometric [Text Word] OR Exercises, Isometric [Text Word] OR Isometric Exercises [Text Word] OR Isometric Exercise [Text Word] OR Exercise, Aerobic [Text Word] OR Aerobic Exercise [Text Word] OR Aerobic Exercises [Text Word] OR Exercises, Aerobic [Text Word] OR Exercise Training [Text Word] OR Exercise Trainings [Text Word] OR Training, Exercise [Text Word] OR Trainings, Exercise [Text Word]) |
| Embase | #1 ‘Colonic Neoplasms’/exp OR ‘Colonic Neoplasms’  #2 ‘Colonic Neoplasm’:ab,kw,ti OR ‘Neoplasm, Colonic’:ab,kw,ti OR ‘Neoplasms, Colonic’:ab,kw,ti OR ‘Colon Neoplasms’:ab,kw,ti OR ‘Colon Neoplasm’:ab,kw,ti OR ‘Neoplasm, Colon’:ab,kw,ti OR ‘Neoplasms, Colon’:ab,kw,ti OR ‘Cancer of Colon’:ab,kw,ti OR ‘Colon Cancers’:ab,kw,ti OR ‘Colon Cancer’:ab,kw,ti OR ‘Cancer, Colon’:ab,kw,ti OR ‘Cancers, Colon’:ab,kw,ti OR ‘Cancer of the Colon’:ab,kw,ti OR ‘Colonic Cancer’:ab,kw,ti OR ‘Cancer, Colonic’:ab,kw,ti OR ‘Cancers, Colonic’:ab,kw,ti OR ‘Colonic Cancers’:ab,kw,ti OR ‘Colon Adenocarcinoma’:ab,kw,ti OR ‘Adenocarcinoma, Colon’:ab,kw,ti OR ‘Adenocarcinomas, Colon’:ab,kw,ti OR ‘Colon Adenocarcinomas’:ab,kw,ti OR ‘CRC’:ab,kw,ti  #3 #1 OR #1  #4 ‘Exercise’/exp OR ‘Exercise’  #5 ‘Exercises’:ab,kw,ti OR ‘Physical Activity’:ab,kw,ti OR ‘Activities, Physical’:ab,kw,ti OR ‘Activity, Physical’:ab,kw,ti OR ‘Physical Activities’:ab,kw,ti OR ‘Exercise, Physical’:ab,kw,ti OR ‘Exercises, Physical’:ab,kw,ti OR ‘Physical Exercise’:ab,kw,ti OR ‘Physical Exercises’:ab,kw,ti OR ‘Acute Exercise’:ab,kw,ti OR ‘Acute Exercise’:ab,kw,ti OR ‘Exercise, Acute’:ab,kw,ti OR ‘Exercises, Acute’:ab,kw,ti OR ‘Exercise, Isometric’:ab,kw,ti OR ‘Exercises, Isometric’:ab,kw,ti OR ‘Isometric Exercises’:ab,kw,ti OR ‘Isometric Exercise ’:ab,kw,ti OR ‘Exercise, Aerobic’:ab,kw,ti OR ‘Aerobic Exercise’:ab,kw,ti OR ‘Aerobic Exercises’:ab,kw,ti OR ‘Exercises, Aerobic’:ab,kw,ti OR ‘Exercise Training’:ab,kw,ti OR ‘Exercise Trainings’:ab,kw,ti OR ‘Training, Exercise’:ab,kw,ti OR ‘Trainings, Exercise’:ab,kw,ti  #6 #4 OR #5  #7 #3 AND #6 |
| Cochrone | #1 MeSH descriptor: [Colonic Neoplasms] explode all trees  #2 (Colonic Neoplasm):ti,ab,kw OR (Neoplasm, Colonic):ti,ab,kw OR (Neoplasms, Colonic):ti,ab,kw OR (Colon Neoplasms):ti,ab,kw OR (Colon Neoplasm):ti,ab,kw OR (Neoplasm, Colon):ti,ab,kw OR (Neoplasms, Colon):ti,ab,kw OR (Cancer of Colon):ti,ab,kw OR (Colon Cancers):ti,ab,kw OR (Colon Cancer):ti,ab,kw OR (Cancer, Colon):ti,ab,kw OR (Cancers, Colon):ti,ab,kw OR (Cancer of the Colon):ti,ab,kw OR (Colonic Cancer):ti,ab,kw OR (Cancer, Colonic):ti,ab,kw OR (Cancers, Colonic):ti,ab,kw OR (Colonic Cancers):ti,ab,kw OR (Colon Adenocarcinoma):ti,ab,kw OR (Adenocarcinoma, Colon):ti,ab,kw OR (Adenocarcinomas, Colon):ti,ab,kw OR (Colon Adenocarcinomas):ti,ab,kw OR (CRC):ti,ab,kw  #3 #1 OR #2  #4 MeSH descriptor: [Exercise] explode all trees  #5 (Exercises):ti,ab,kw OR (Physical Activity):ti,ab,kw OR (Activities, Physical):ti,ab,kw OR (Activity, Physical):ti,ab,kw OR (Physical Activities):ti,ab,kw OR (Exercise, Physical):ti,ab,kw OR (Exercises, Physical):ti,ab,kw OR (Physical Exercise):ti,ab,kw OR (Physical Exercises):ti,ab,kw OR (Acute Exercise):ti,ab,kw OR (Acute Exercise):ti,ab,kw OR (Exercise, Acute):ti,ab,kw OR (Exercises, Acute):ti,ab,kw OR (Exercise, Isometric):ti,ab,kw OR (Exercises, Isometric):ti,ab,kw OR (Isometric Exercises):ti,ab,kw OR (Isometric Exercise):ti,ab,kw OR (Exercise, Aerobic):ti,ab,kw OR (Aerobic Exercise):ti,ab,kw OR (Aerobic Exercises):ab,kw,ti OR (Exercises, Aerobic):ti,ab,kw OR (Exercise Training):ti,ab,kw OR (Exercise Trainings):ti,ab,kw OR (Training, Exercise):ti,ab,kw OR (Trainings, Exercise):ti,ab,kw  #6 #4 OR #5  #7 #3 AND #6 |
| Web of Knowledge | TS=(Colonic Neoplasms OR Colonic Neoplasm OR Neoplasm, Colonic OR Neoplasms, Colonic OR Colon Neoplasms OR Colon Neoplasm OR Neoplasm, Colon OR Neoplasms, Colon OR Cancer of Colon OR Colon Cancers OR Colon Cancer OR Cancer, Colon OR Cancers, Colon OR Cancer of the Colon OR Colonic Cancer OR Cancer, Colonic OR Cancers, Colonic OR Colonic Cancers OR Colon Adenocarcinoma OR Adenocarcinoma, Colon OR Adenocarcinomas, Colon OR Adenocarcinomas OR CRC) AND TS=(Exercise OR Exercises OR Physical Activity OR Activities, Physical OR Activity, Physical OR Physical Activities OR Exercise, Physical OR Exercises, Physical OR Physical Exercise OR Physical Exercises OR Acute Exercise OR Acute Exercise OR Exercise, Acute OR Exercises, Acute OR Exercise, Isometric OR Exercises, Isometric OR Isometric Exercises OR Isometric Exercise OR Exercise, Aerobic OR Aerobic Exercise OR Aerobic Exercises OR Exercises, Aerobic OR Exercise Training OR Exercise Trainings OR Training, Exercise OR Trainings, Exercise) |
