# Supplementary material for: Physical exercise as a potential adjuvant therapy: effects on inflammation and nutrition in colorectal cancer patients—a systematic review and meta-analysis
Source: Front Nutr. 2025 Jun 26;12:1612674. doi: 10.3389/fnut.2025.1612674 (PMC12243031; doi:10.3389/fnut.2025.1612674)
Supplement: Supplementary file 2 [file Table_2.docx]

Table S2 PICOS framework

| describe | explain |
| --- | --- |
| populations | Colorectal cancer patients |
| interventions | physical exercise (at least 4 weeks’ duration of intervention, comprised aerobic exercise, or resistance exercise or their combination) |
| comparators | Usual care |
| outcomes | Nutrition indicators (BMI, body weight, body fat, waist circumference), inflammation marker (CRP, IL-6, TNF-α), |
| study designs | Selected relevant studies published between January, 1945, and June, 2024, by searching PubMed Central, Embase, Cochrane, and Web of Knowledge on June 17, 2023, respectively. In addition, ongoing clinical trials about "physical exercise for CRC patients" were searched on the international clinical trial registry (http://clinicaltrials.gov/) as other sources.  All the outcomes indicator were presented as the mean ± standard deviation (SD), and for the data in the form of median and interquartile range (IQR), these would be counted after determining whether the data were skewed and then converted by the formula to get the form of mean ± SD.  Assessed the risk for bias according to the PRISMA using the Cochrane Risk of Bias tool. The quality of available evidence was assessed using the GRADEpro GDT tool. |
