# Supplementary material for: Physical exercise as a potential adjuvant therapy: effects on inflammation and nutrition in colorectal cancer patients—a systematic review and meta-analysis
Source: Front Nutr. 2025 Jun 26;12:1612674. doi: 10.3389/fnut.2025.1612674 (PMC12243031; doi:10.3389/fnut.2025.1612674)
Supplement: Supplementary file 3 [file Image_1.pdf]

	Random sequence generation (selection bias)	Allocation concealment (selection bias)	Blinding of participants and personnel (performance bias)	Blinding of outcome assessment (detection bias)	Incomplete outcome data (attrition bias)	Selective reporting (reporting bias)	Other bias
Abrahamson, Page E. 2007	+	+	+	+	+	+	+
AhnAhn, K. Y. 2013	+	?	+	+	+	+	+
Bousquet-Dion 2018	+	+	+	+	+	+	+
Brown, J. C. 2023	+	?	−	+	+	−	+
Campbell, K. L. 2008	+	?	+	+	+	+	+
Carli, Francesco 2020	+	+	+	+	+	+	+
Devin, J. L. 2019	?	?	+	−	+	+	+
Lee, M. K. 2017	+	+	?	?	+	+	+
Ligibel, J. A. 2012	+	?	+	+	+	+	+
Lin, K. Y. 2014	−	?	+	+	+	+	+
Min, J. 2023	+	+	−	+	+	+	+
Møller, T. 2015	+	+	+	−	+	+	−
Toffoli, E. C. 2021	+	?	?	?	+	+	+
Van Blarigan, E. L. 2022	+	+	+	+	+	+	?
Van Vulpen, J. K. 2016	+	+	−	+	+	+	+

Fig. S1 risk of bias summary.
