# Supplementary figures and images for: Physical exercise as a potential adjuvant therapy: effects on inflammation and nutrition in colorectal cancer patients—a systematic review and meta-analysis
Source: Front Nutr. 2025 Jun 26;12:1612674. doi: 10.3389/fnut.2025.1612674 (PMC12243031; doi:10.3389/fnut.2025.1612674)

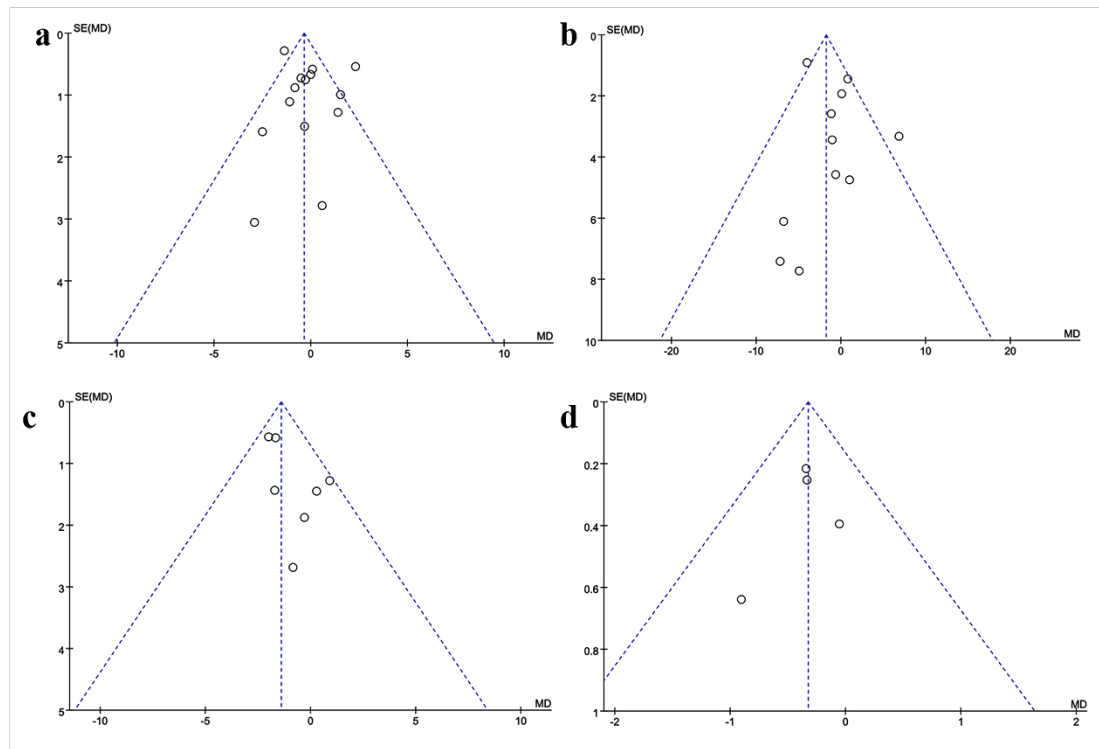

Fig. S2 Funnel plot of (a) BMI; (b)Body weight; (c)Body fat; (d)CRP.

Supplement: Supplementary file 4 [file Image_2.pdf]

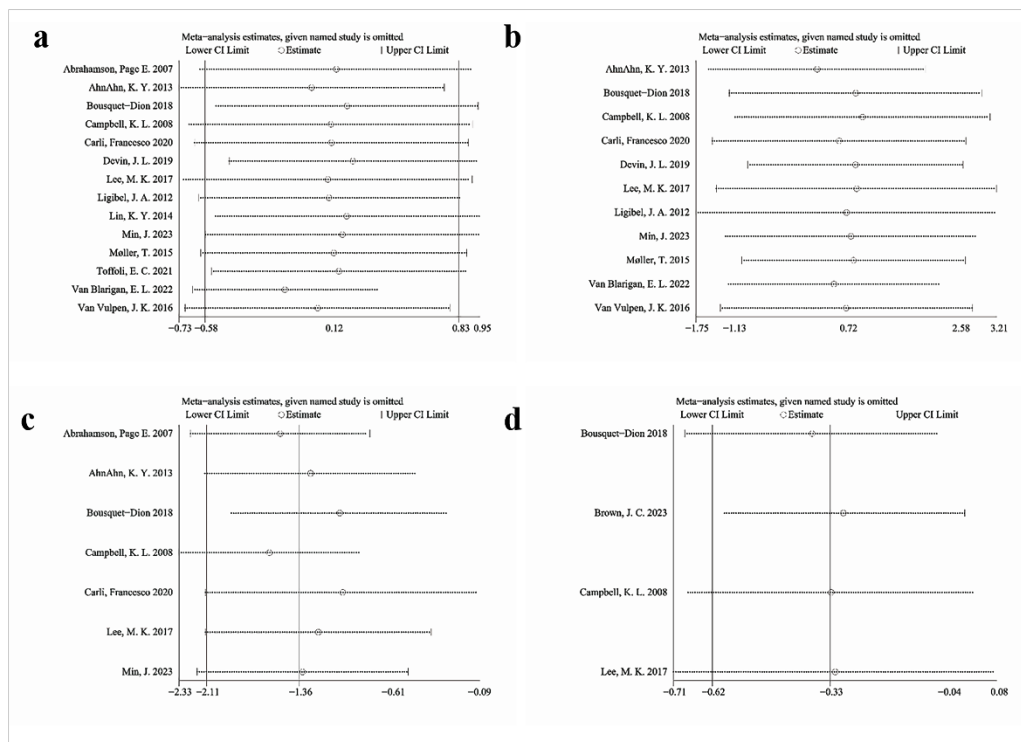

Fig.S3 Sensitivity analysis of (a)BMI; (b)Body weight; (c)Body fat; (d)CRP.

Supplement: Supplementary file 5 [file Image_3.pdf]
