# Supplementary material for: Physical exercise as a potential adjuvant therapy: effects on inflammation and nutrition in colorectal cancer patients—a systematic review and meta-analysis
Source: Front Nutr. 2025 Jun 26;12:1612674. doi: 10.3389/fnut.2025.1612674 (PMC12243031; doi:10.3389/fnut.2025.1612674)
Supplement: Supplementary file 7 [file Image_5.pdf]

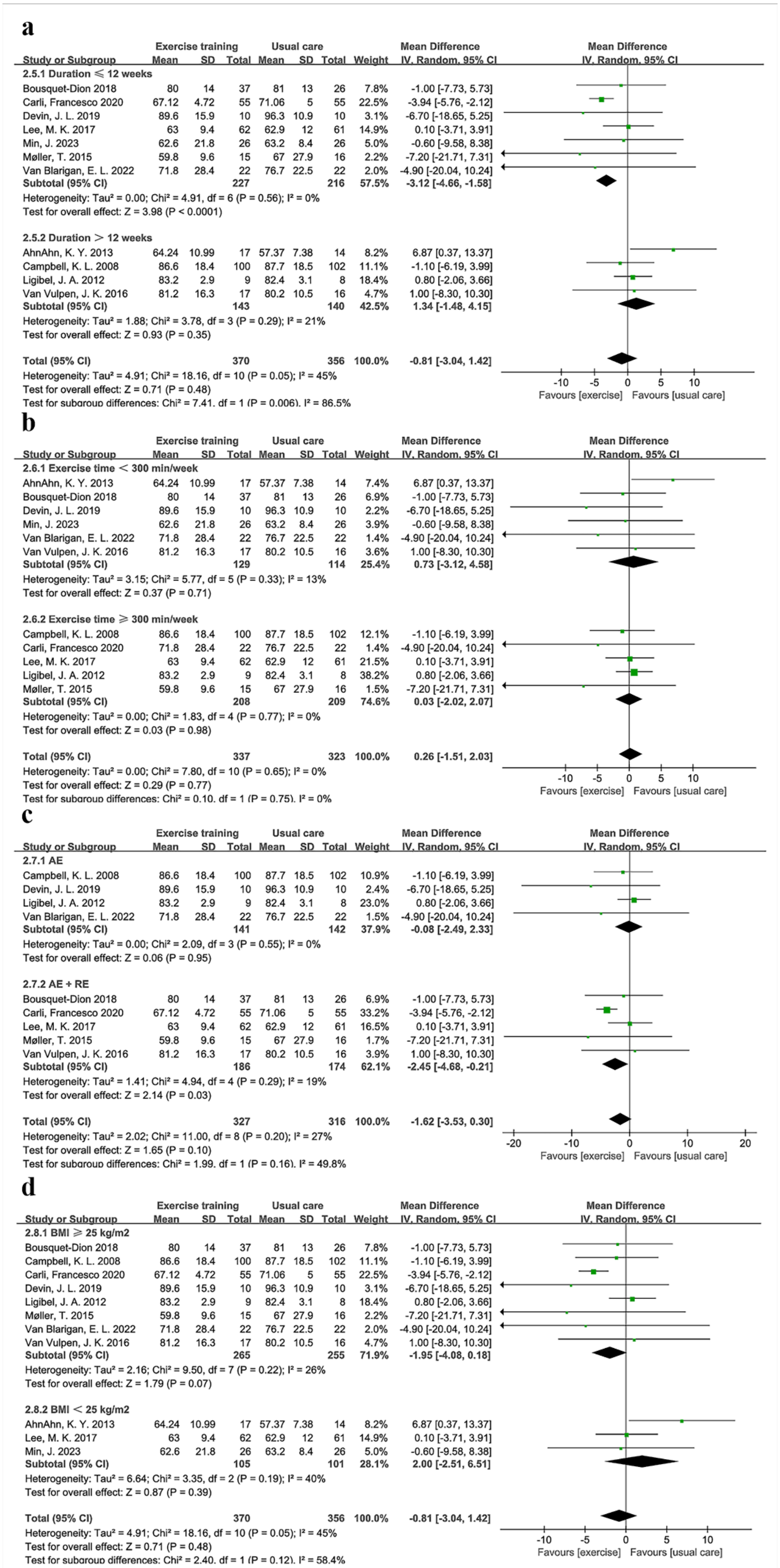

Fig.S5 Subgroup analysis of Body weight with different (a) exercise duration; (b)exercise time; (c) exercise type and (d)baseline BMI.
