# Supplementary material for: Physical exercise as a potential adjuvant therapy: effects on inflammation and nutrition in colorectal cancer patients—a systematic review and meta-analysis
Source: Front Nutr. 2025 Jun 26;12:1612674. doi: 10.3389/fnut.2025.1612674 (PMC12243031; doi:10.3389/fnut.2025.1612674)
Supplement: Supplementary file 9 [file Image_7.pdf]

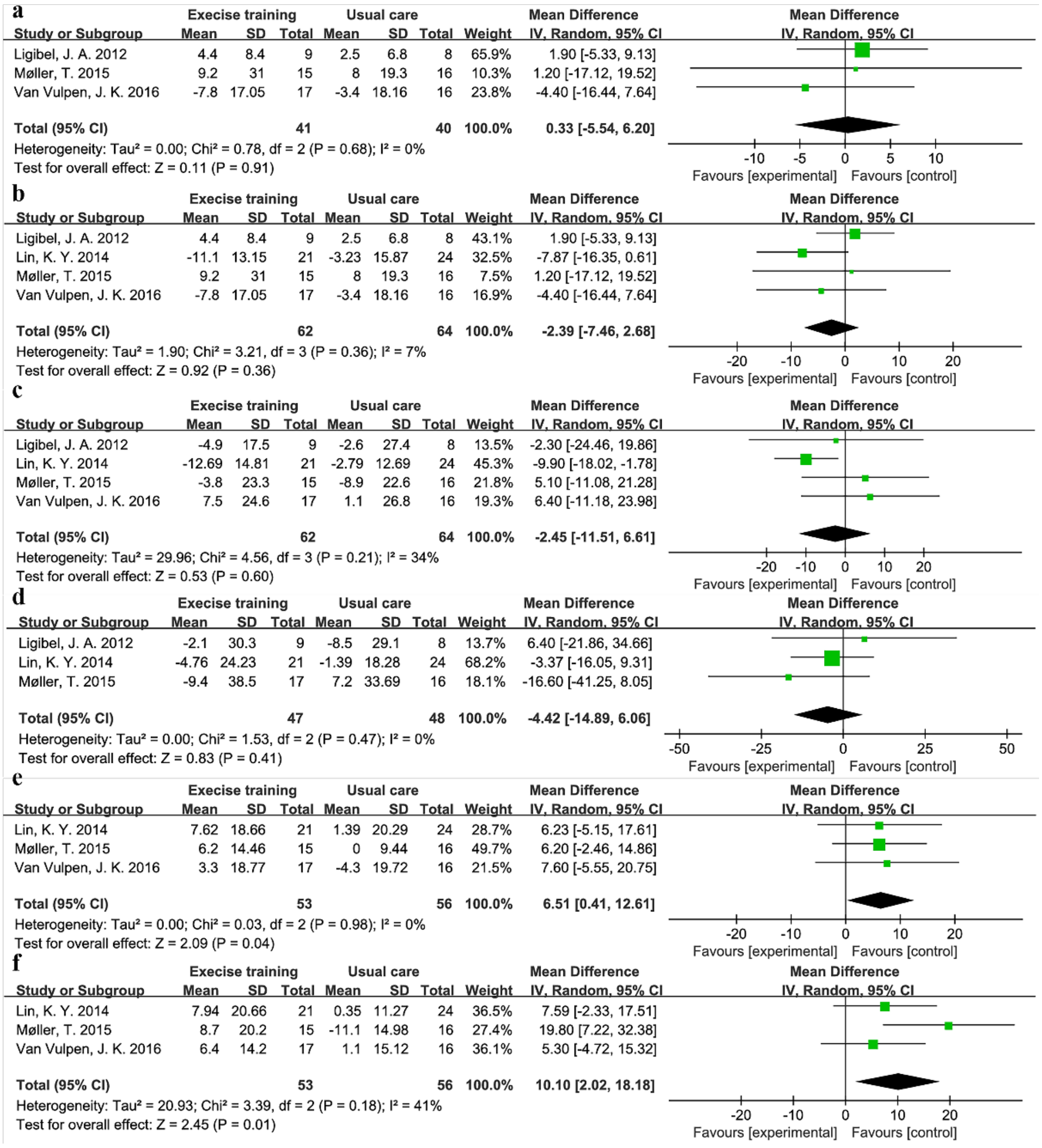

Fig.S7 Forest plot of the secondary results, a quality of life; b Fatigue; c Pain; d Insomnia; e Physical functioning; f Emotional functioning.
